# Research Progress of Sirtuin4 in Cancer

**DOI:** 10.3389/fonc.2020.562950

**Published:** 2021-01-08

**Authors:** Yibing Bai, Jiani Yang, Ying Cui, Yuanfei Yao, Feng Wu, Caiqi Liu, Xiaona Fan, Yanqiao Zhang

**Affiliations:** ^1^ Department of Gastrointestinal Medical Oncology, Harbin Medical University Cancer Hospital, Harbin, China; ^2^ Translational Medicine Research and Cooperation Center of Northern China, Heilongjiang Academy of Medical Sciences, Harbin, China; ^3^ Department of Radiation Oncology, Harbin Medical University Cancer Hospital, Harbin, China; ^4^ Department of Gastroenterology, The First Affiliated Hospital of Harbin Medical University, Harbin, China

**Keywords:** sirtuin4, cancer, structure, localization, enzymatic activities, metabolism, mitochondria

## Abstract

Sirtuins (SIRTs) are members of the silent information regulator-2 family. They are a conserved family of nicotinamide adenine dinucleotide-dependent protein lysine deacylases. SIRTS are involved in intricate cellular processes. There are seven subtypes of SIRTs (1–7) in mammals. SIRT4 is located mainly in mitochondria and has various catalytic activities. These enzyme activities give it a diverse range of important biologic functions, such as energy metabolism, oxidative stress, and aging. Cancer is characterized as reprogramming of energy metabolism and redox imbalance, and SIRT4 can affect tumorigenesis. Here, we review the structure, localization, and enzyme activity of SIRT4 and its role in various neoplasms.

## Introduction

Cancer has become the first or second most prevalent cause of premature death in ~100 countries (1). Cancer occurrence is closely related to genomic instability, which enables cells to maintain proliferation signals, escape growth inhibitors, avoid immune damage, resist cell death and, eventually, develop into a life-threatening malignant tumor. Hanahan et al. described the “hotspots” and new advances in oncology during the last decade and proposed new tumor features: avoidance of immune destruction, promotion of tumor inflammation, abnormal cell energy, genomic instability, and mutations (2). In recent years, a new feature of malignant tumors has attracted increasing attention: the reprogramming of energy metabolism (3).

Sirtuin4 (SIRT4) belongs to sirtuins (SIRTs). The latter is a family of highly conserved nicotinamide adenine dinucleotide (NAD^+^)-dependent enzymes that regulate lifespan, ageing and metabolism (4). Mammalian SIRTs are considered class-III histone deacetylases (HDACs). According to sequence homology, HDAC enzymes can be divided into three types (5, 6). Class I contains HDAC1, 2, 3, and 8, all of which are homologs of the RPD3 protein in yeast. Class II contains HDAC4, 5, 6, 7, 9, and 10, all of which are homologs of the yeast Hda1 protein. While class III contains seven SIRT homologs of the silent information regulator 2 (Sir2) protein of yeast (7).

SIRT4 is located mainly in the mitochondria of cells. SIRT4 has adenosine diphosphate (ADP)-ribosyltransferase activity and can deacetylate (8, 9). In recent years, it has been found that SIRT4 catalyzes removal of lipoyl- and biotinyl-lysine modifications more efficiently than deacetylate (10). SIRT4 also has lysine-deacylase activity for removing 3,3-dimethylsuccinyl (DMS), 3-hydroxy-3-methylpentadienyl (HMG), 3-methylglutaryl (MG) and 3-methylglutamyl (MGc) (11, 12). These enzyme activities enable SIRT4 to regulate the targets of glutamate dehydrogenase (GDH), malonyl-CoA decarboxylase and the pyruvate dehydrogenase complex (PDHC) directly. In this way, SIRT4 plays an irreplaceable part in the metabolic regulation of glutamine, lipids, and glucose in malignant tumors (8–10).

In this review, we summarize the structure and localization, enzyme activity, and role of SIRT4 in malignant tumors.

## Structure and Location of SIRT4

### Structure

SIRT4 is a protein of size 35.2 kDa located on chromosome 12q24.31 (13). According to sequencing and analyses of its primary structure, SIRT4 has essential amino acids that are involved in the deacylase reaction. SIRTs have a conserved catalytic core of ~275 amino acids flanked by N-terminal and C-terminal sequences of variable length. The small zinc-binding domain and large Rossmann-folding domain enable all SIRTs to bind specifically to NAD^+^ as a cofactor (14). SIRT4 contains a homologous SIRTs deacylase domain, a conserved catalytic histidine (H161), substrate specificity, as well as a Zn^2+^-binding and a Rossmann fold/NAD^+^-binding motif (amino acids 62–82, 143–146, 260–262, 286–288). The main sequencing analysis and alignment of three types of mitochondrial SIRTs (SIRT3, SIRT4, and SIRT5) have shown that the position of amino acids is very important for mitochondrial localization, NAD^+^ binding, catalysis, substrate specificity, and Zn^2+^ binding (12).

The secondary structure of SIRT4 contains α-helices and β-sheets, which are at virtually the same location as those in other mitochondrial SIRTs (SIRT3 and SIRT5). The evolutionarily conserved α-helices of the SIRT4 catalytic pocket can confer specific interactions with negatively charged lysine molecules. Comparison of the crystal structure of SIRT4 with that of other SIRTs has revealed that the most significant difference is an extended ~12-residue SIRT4 loop (residues 195–206) between α8 and α9 in the Zn^2+^-binding module. This loop is oriented deep into the catalytic core and contributes to the lining of the active site. Specifically, this loop is not present in any other SIRT isotypes, so it is called the “SIRT4 loop”. The latter folds into active sites to potentially regulate catalysis. Another unusual feature of the catalytic core of SIRT4 is the channel that branches from the acyl-lysine binding channel to the protein surface. It can accommodate longer substrate acyl groups, and may act as a binding site that regulates metabolites, which rationalizes its activities. This channel is conservative in SIRT4, but is different in other isotypes. However, the specific function of this SIRT4 channel has yet to be studied deeply (11).

Phylogenetic analyses can reveal the patterns of evolutionary stress of specific amino acids, which may be important for protein function. Phylogenetic analyses of 5869 SIRTs domains of proteins from 3,562 species are consistent with studies which divided human SIRTs into four categories: SIRT1–3 occupy class I, SIRT4 occupies class II, SIRT5 occupies class III, and SIRT6 and SIRT7 occupy class IV (12).

### Localization

SIRT4 is distributed in humans in the heart, brain, liver, kidney, muscle, pancreas, thymus, lung, spleen, bone marrow, uterus, ovary, and testis (9, 15–18). The wide distribution of SIRT4 may indicate the importance of its biologic functions.

Upon fusion of SIRTs with green fluorescent protein (GFP), SIRT4-GFP co-localizes with the mitochondrial-specific probe MitoFluor™ as well as the mitochondrial labeler MitoTracker™. SIRT4 has a N-terminal mitochondrial targeting sequence (MTS) of length of 28 amino acids. This MTS is responsible for localization of this enzyme in the mitochondrial cavity (15). Studies have shown that SIRT4-EGFP and SIRT4-FLAG are co-located with mitochondrial markers, and that SIRT4 is closely related to the metabolic activity and significant biologic functions of mitochondria (9, 10, 15, 19). Moreover, mitochondria are responsible for oxidative metabolism through the Krebs cycle, β-oxidation of fatty acids (FAs) and oxidative phosphorylation, which result in production of most cellular adenosine 5′ triphosphate (ATP) (20). Therefore, there is an association between SIRT4 and energy metabolism.

Recently, it was shown that SIRT4 exists not only in mitochondria but also in the cytoplasm and nucleus (21). Fusion of SIRT4 with a very mature fluorescent protein (FP) variant to form a “super folder GFP” (sfGFP) can enable localization and visualization of *de novo* protein synthesis dynamically (22, 23). Even at very low expression, SIRT4-sfGFP was not fully introduced into mitochondria and accumulated in the cytoplasm and nucleus. Moreover, SIRT4-sfGFP accumulated in the outer mitochondrial membrane (OMM) but did not enter the mitochondrial matrix. However, the fast-folding sfGFP fused with mature MTS to be introduced into the mitochondrial cavity. Those findings suggest that SIRT4 enters mitochondria slowly. Moreover, SIRT4-sfGFP accumulation in the OMM is related to the swelling of mitochondria, which indicates the toxicity of this specific fusion construct (21).

Another maneuver is based on “split FP technology” for studying introduction of proteins into the mitochondrial matrix (24). To avoid the mis-localization and mitochondrial swelling of “clunky” SIRT4-FP fusion constructors, FP splitting based on sfCherry2 self-complementarity was used. Using this established method, SIRT4-sfCherry2_11_ was introduced and located in the mitochondrial matrix (25). Furthermore, the complementary sfCherry2_1–10_ constructors targeting the mitochondrial cavity and nucleus were co-expressed. This new trigonal probe was named “mitochondrial sirtuin 4 tripartite abundance reporter” (mito-STAR), which realized the visualization of SIRT4 introduction into the mitochondrial matrix and nucleus. Using mito-STAR, it was found that, although the mitochondrial targeting of SIRT4-sfCherry2_11_ had been improved significantly compared with that using SIRT4-sfGFP, many cells showing nuclear fluorescence signals could be detected, which suggested the nuclear abundance of SIRT4-sfCherry211 under a dormant condition (21).

During the mitochondrial unfolded protein response (mtUPR), the stress state caused by accumulation of unfolded proteins in mitochondria and accumulation of mitochondrial-targeted proteins in the cytoplasm and nucleus contributes to a retrograde signaling pathway (26, 27). It has been reported that SIRT3 participates in mtUPR (28, 29). When mito-STAR was applied under mitochondrial stress, SIRT4 expression in the nucleus was increased significantly (21). Once again, the idea that SIRT4 promotes retrograde signals from mitochondria to the nucleus was supported by data.

Consistently, it has been found that a fraction of endogenous or ectopically expressed SIRT4 is present in the cytosol and localizes predominantly to centrosomes. Confocal spinning disk microscopy has revealed that SIRT4 is found during the cell cycle dynamically at centrosomes with an intensity peak in the G2 phase and early mitosis. Moreover, SIRT4 precipitates with microtubules and interacts with structural (α, β-tubulin, γ-tubulin, TUBGCP2, TUBGCP3) and regulatory (HDAC6) microtubule components as detected by co-immunoprecipitation experiments and mass spectrometry of the mitotic SIRT4 interactome. SIRT4 overexpression resulted in a pronounced decrease in the level of acetylated α-tubulin (K40) associated with altered microtubule dynamics in mitotic cells. Importantly, the N-terminally truncated variant SIRT4 (ΔN28), which cannot translocate into mitochondria, delayed mitotic progression and reduced cell proliferation. Hence, SIRT4 may exert its anti-proliferative role through mitochondrial/metabolism-dependent and mitochondria-independent functions (30).

## Enzyme Activity of SIRT4

### Deacetylase Activity

All SIRTs have conserved deacetylase domains. Hence, these enzymes are usually called “lysine deacetylases” which, in contrast to acetyltransferases, can deacetylate from lysine residues (31). However, SIRTs show different catalytic efficiency for this modification. Initial studies revealed scarce deacetylase activity in SIRT4 using histone substrates or substrates of bovine serum albumin (9, 18, 32). Nevertheless, the lack of deacetylase activity in SIRT4 may be due to the absence of a suitable substrate, German et al. showed, for the first time, that SIRT4 has substrate-specific deacetylase activity. SIRT4 controls lipid catabolism through deacetylation of malonyl-CoA decarboxylase (8). In addition, SIRT4 has been shown to have significant deacetylation activity on acetylome peptide microarrays, thereby revealing the physiologic candidates for deacetylation. MtHsp60 (Lys31) and Stress-70 (Lys135, 595) have the highest hit rates, which are related to the proliferation and senescence of cells, and also involve NAD^+^ transhydrogenase (Lys394, 331, 397), which is important for to responses redox stress (33). Recently, it has been shown that in pancreatic ductal adenocarcinoma (PDAC), SIRT4 could deacetylate branched-chain amino acid transaminase 2 (BCAT2) at lysine 44 (K44), and that BCAT2 was stabilized by SIRT4 deacetylation, which promoted catabolism of branched-chain amino acids, cell proliferation and the growth of pancreatic tumors (34).

### ADP-Ribosyltransferase Activity

SIRT4 ADP-ribosylates GDH to repress its activity using NAD in pancreatic β-cell mitochondria. In this way, it prevents glutamine being an insulin secretagogue and down-regulating insulin secretion in response to amino acids. This effect has been shown to be alleviated during calorie restriction. During calorie restriction, the ratio of NAD/NADH decreased, SIRT4 activity decreased, and GDH activity increased, thereby up-regulating insulin secretion in response to glutamine and leucine (9). Ahuja and colleagues showed that SIRT4 has a highly efficient ADP-ribosyltransferase function for histone and bovine serum albumin (18). However, some scholars believe that the ADP-ribosyltransferase activity of SIRT4 may be due to the inefficient side-effects of deacetylase activity, and to be unrelated to physiology (35). Therefore, the role of the ADP-ribosyltransferase activity of SIRT4 merits further study.

### Removal of Lipoyl and Biotinyl Lysine Modifications

SIRT4 can inhibit the activity of PDHC components by hydrolyzing lipoamide cofactors from dihydrolipoyllysine-residue acetyltransferase (DLAT) E2 component of the PDHC. SIRT4 shows enzymatic activity only in the presence of NAD^+^. To compare the relative preference of SIRT4 for different acyl lysine peptides, ion chromatography was used to quantify the percentage of unmodified peptides produced by each substrate. SIRT4 showed the highest efficacy for removing the lipoyl modification. The relative amount of unmodified product formed after reacting with SIRT4 was 11% for lipoyl, 3% for biotinyl, and 0.3% for acetyl. Moreover, compared with other mitochondrial SIRTs, SIRT4 had the highest catalytic efficiency for lipamide modification (10).

### Other Activities

To screen the activity of recombinant SIRT4 against various acyl histone peptides, SIRT4 displayed dedecanoylase and deoctanoylase activity, as well as exhibited low (but measurable) activity against lipoyl, succinyl, and dodecanoyl substrates (36).

Testing SIRT4 from an acyl library of carbamoyl phosphate synthetase 1 (CPS1)-Lys527 peptides showed a specific profile in monitoring of nicotinamide (NAM) release from NAD^+^ by conjugated enzyme assays. Testing revealed that use of lipoylated and biotinylated substrates could enhance activity further, but the highest activity was observed with DMS substrates (eight-fold stronger than deacetylation). DMS was compared with the physiologically occurring acyl part of activated coenzyme A (CoA)-thioesters to potentially modify the sidechains of lysine (37, 38), and the HMG group was found to be the most relevant. HMG-modified CPS1-Lys527 peptides elicited SIRT4 activity that was similar to that of the DMS-CPS1 substrate, and approximately three-fold higher than that of the acetyl peptide (11).

**Figure 1:**
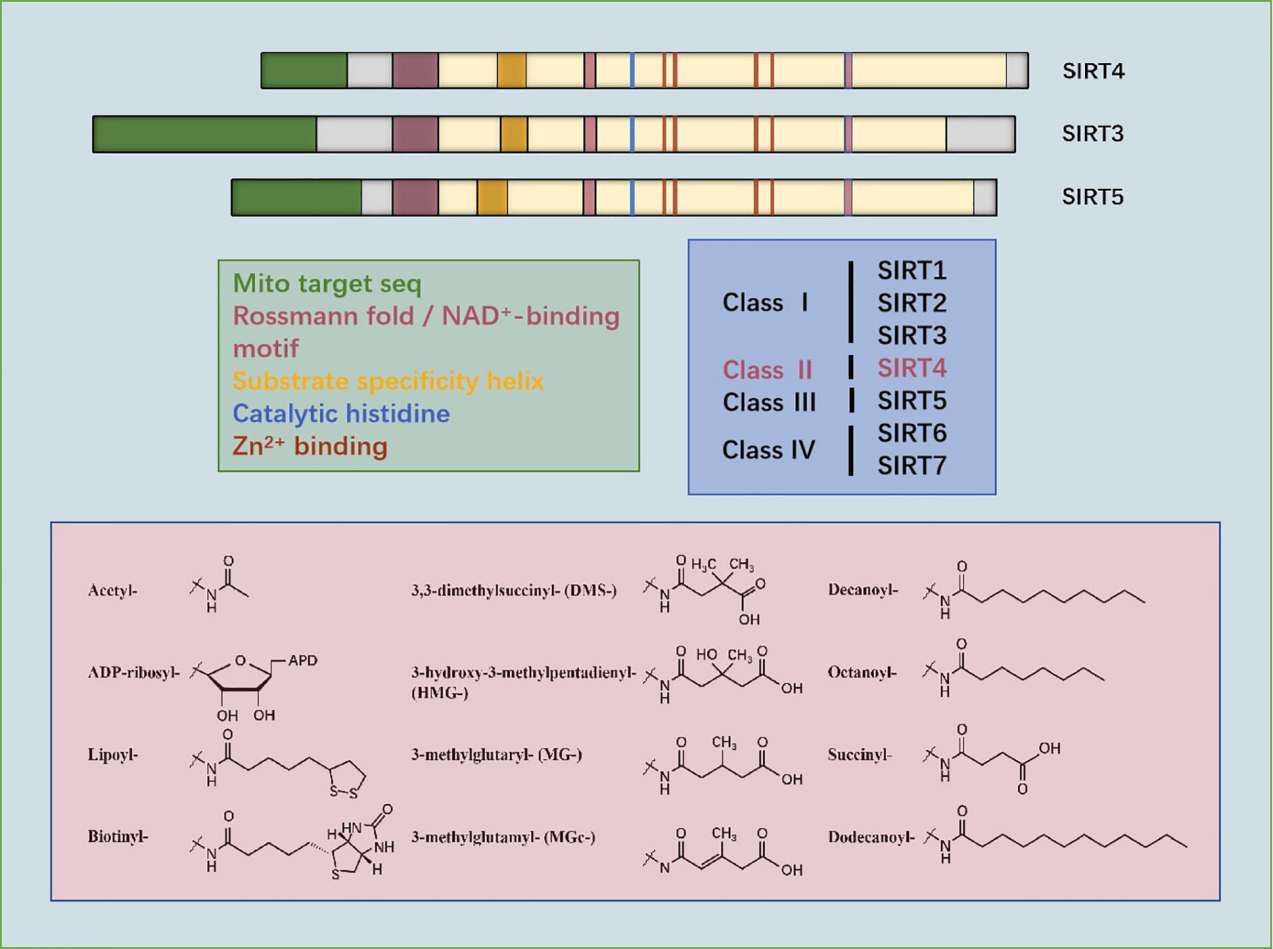
Sequencing of amino acids and mitochondrial localization, NAD+-binding, substrate specificity, catalytic, histidine- and Zn2+-binding domains of SIRT3, SIRT4, and SIRT5. Phylogenetic analyses show that SIRT4 occupies class II. Structures of the substrates catalyzed by SIRT4 are shown.

Anderson and colleagues used a combination of phylogeny, structural biology, and enzymology to demonstrate that SIRT4 catalyzes removal of HMG, as well as the associated modified MG and MGc, from the lysine residues of the target protein. Moreover, they found that the metabolites responsible for these post-translational modifications were intermediates of leucine oxidation, and that SIRT4 removed these modifications to restore leucine catabolism. In addition, disorders in leucine metabolism in SIRT4 knockout (KO) mice led to increased basal and stimulated insulin secretion, which developed gradually into glucose tolerance and insulin resistance. Those findings confirmed the strong enzyme activity of SIRT4, revealed the mechanism of controlling fluxes in branched-chain amino acids, and positioned SIRT4 as a key factor in maintaining glucose homeostasis during insulin secretion and senescence (12) (**Figure 1**).

## Role of SIRT4 in Cancer

Analyses of data from The Cancer Genome Atlas (TCGA) database demonstrated that SIRT4 expression was downregulated in 30 cancer types. Similarly, compared with samples of normal tissue, 19,120 human-cancer tissues also showed reduced expression of SIRT4, which were analyzed from three datasets (GTEX, TARGET, TCGA). Wang and colleagues studied the copy number of SIRT4 in various cancer types using the Tumorscape database, and found that SIRT4 was located in the region in chromosome 12 that was deleted significantly in the whole dataset (39).

Through a comprehensive meta-analysis of SIRT4 expression in human tumors, it was found that expression of SIRT4 mRNA in tissues with bladder, breast, colon, gastric, ovarian, or thyroid cancer was significantly lower than that in corresponding normal tissues. Moreover, the loss of SIRT4 expression showed a strong association with short-term metastasis in breast-cancer patients. In addition, SIRT4 overexpression led to tumorigenesis and inhibited tumor development in a xenotransplantation model using Tsc2^−/−^ mouse embryonic fibroblasts (MEFs) (40).

Fu and colleagues employed immunohistochemical (IHC) staining to measure SIRT4 expression in lung, ovarian, pancreatic, hepatocellular, bladder, prostate, or renal cancer tissues. Compared with the positive staining of the corresponding normal adjacent tissues, SIRT4-negative or weak staining was observed in most tumor types (41).

A lack of SIRT4 accelerates the morbidity of spontaneous tumors throughout life. Jeong and colleagues found that several types of neoplasms were incurred in ~63% (22/35) of SIRT4-KO animals, whereas 24% (6/25) of wild-type (WT) littermates had tumors. At the age of 18−26 months, more SIRT4-KO mice (45.5%) developed lung tumors than WT mice (8.3%). Also, 41% (7/17) of these lung tumors were adenomas and 59% (10/17) were carcinomas, from SIRT4-KO mice. In addition, 60% of female SIRT4-KO mice had cystic endometrial hyperplasia, whereas 15% of WT mice showed mild endometrial hyperplasia (42).

**Figure 2:**
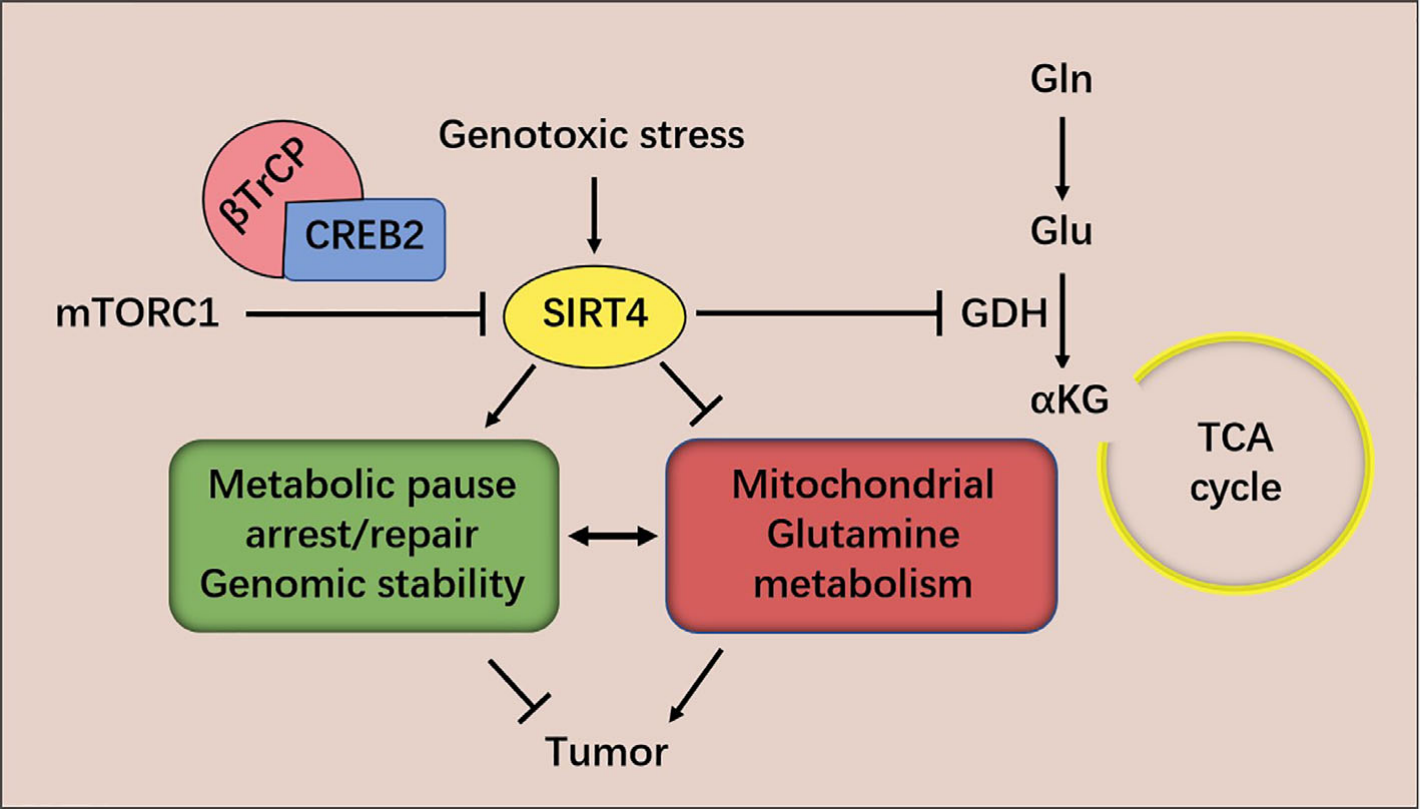
mTORC1 regulates SIRT4 expression and glutamine metabolism. SIRT4 inhibits glutamine metabolism in mitochondria after DNA damage and regulates cell-cycle progression and genomic fidelity in response to DNA damage.

The evidence provided above suggests that SIRT4 is a tumor suppressor. Two mechanisms have been proposed by which SIRT4 inhibits tumorigenesis and tumor development, as studied in MEFs. The first hypothesis is based on the observation that genomic instability and metabolic changes are key features of many cancer cells (43, 44). DNA damage triggers a closely coordinated signal-transduction response to maintain genomic integrity by promoting cell-cycle arrest and DNA repair (45, 46). It has been discovered that DNA damage triggers a key barrier to glutamine metabolism, which is necessary for an appropriate response to DNA damage. This blockade requires mitochondrial SIRT4, which is induced by various genotoxic proxies and inhibits glutamine metabolism into the tricarboxylic acid (TCA) cycle. SIRT4 can inhibit glutamine metabolism in mitochondria after DNA damage and regulate cell-cycle progression and genomic fidelity in response to DNA damage. Loss of SIRT4 leads to increased glutamine-dependent proliferation and stress-induced genomic instability, which results in a carcinogenic phenotype. Therefore, SIRT4 plays an important part in regulating glutamine metabolism and genomic instability (42). The second hypothesis is based on the observation that atypical activation of serine/threonine kinase in mammalian target of rapamycin complex 1 (mTORC1) controls GDH activity by inhibiting SIRT4 expression, thereby stimulating glutamine uptake, not only in MEFs, but also in human epithelial tumor cell lines [colon cancer (DLD1) and prostate cancer (DU14)]. The mTORC1 pathway controls SIRT4 negatively by promoting proteasome-mediated degradation of cyclic adenosine monophosphate response element binding (CREB)-2. The latter is recognized as a transcription factor of SIRT4. mTORC1 activation promotes the binding of CREB2 to E3 ubiquitin ligase βTrCP and regulates the ubiquitin-based degradation of CREB2 (40) (**Figure 2**).

### Neoplasms of the Digestive System

#### Hepatocellular Carcinoma (HCC)

SIRT4 expression is reduced in human HCC. The genomic mutation, amplification, and mRNA expression of SIRT4 in HCC were analyzed using the cBioPortal tool of cancer genomics (www.cbioportal.org/). As a result, there was almost no mutation or amplification of SIRT4, and down-regulation of mRNA expression was found in 5.23% of human HCC samples, which could be explained by frequent and deep deletions of the locus. Also, down-regulation of SIRT4 expression was detected in 83 individual HCC datasets compared with that in matched normal tissues. SIRT4 expression was decreased in early stage (I–II) HCC, especially during cancer progression, indicating that downregulation of SIRT4 expression may be necessary for the initiation and maintenance of tumors. By measuring SIRT4 expression in 364 HCC samples, it was found that low expression of SIRT4 was associated with shorter recurrence time. Moreover, SIRT4 deficiency promoted the proliferation and migration of HCC cells *in vitro* and promoted tumorigenesis and lung metastasis of HCC *in vivo* (39).

Unbiased gene set enrichment analysis revealed that SIRT4 defection was related to activation of mTOR signal transduction. SIRT4 deletion increased expression of the downstream genes of mTOR (MYC, CCND1, HIF1A, and SREBP1) rather than expression of mTOR itself. Consistently, expression of the upstream inhibitors of mTOR (ULK1, TP53, and CDKN1A) was decreased. In addition, IHC analyses and western blotting exhibited that SIRT4-KO increased mTOR phosphorylation, further implying that SIRT4 ablation mediates the activity of mTOR rather than its transcription. Rapamycin (classical inhibitor of mTOR) diminishes mTOR activation intensely, as well as the consequent hyper-proliferative phenotypes aroused by SIRT4 ablation *in vitro* and *in vivo*. More importantly, rapamycin is almost undetectable in control HCC cells with sufficient SIRT4 expression, which may help to explain the resistance to mTOR-targeted therapy for HCC (39).

Tumor cells are characterized by rapid proliferation which, in some scenarios, is driven by increased utilization of glutamine (47). As mentioned above, SIRT4 restricts glutamine utilization to inhibit TCA circulation in response to DNA damage (42). In HCC, SIRT4 depletion also leads to a remarkable increase in glutamine uptake and NH4^+^ production and, importantly, glucose uptake and production of lactic acid are not affected. Hence, SIRT4 loss fortifies the ability of cells to utilize glutamine. As a metabolic sensor, adenosine monophosphate-activated protein kinase (AMPK) inhibits metabolic reprogramming of glycolysis and biosynthesis pathways in cancer by regulating expression of hypoxia-inducible factor 1α (HIF1α) or mTOR (48, 49). Studies have shown that SIRT4 ablation reduces the phosphorylation of AMPKα and its target acetyl-CoA carboxylase (ACC) significantly. As a result, it was observed that mTOR activation increased through phosphorylation of the downstream effectors of TORC1: S6K and 4E-BP1. In addition, SIRT4 status had no effect on the gene expression of AMPK, ACC, S6K, or 4E-BP1. Interestingly, AMPK deficiency restored the glutamate reduction caused by SIRT4 overexpression. Taken together, those results suggest that SIRT4 deletion enhances the metabolic flux of glutamyl conversion, which is combined with AMPK inactivation in HCC cells. Moreover, AMPK is activated by the tumor suppressor liver kinase B1 (LKB1), a serine/threonine kinase that phosphorylates AMPK at high levels of AMP (50). Silencing expression of LKB1 or AMPKα counteracts the effects of SIRT4 depletion or overexpression, respectively, which demonstrates that SIRT4 inhibits the mTOR pathway through a mechanism that is dependent upon LKB1 and AMPKα (39).

The anti-hyperglycemia drug metformin has been shown to inhibit expression of mitochondrial complex I (51). This action leads to an increase in expression of intracellular AMP and ADP, which bind to the gamma regulatory subunit of AMPK and promote LKB1-mediated AMPK phosphorylation (52). After treatment with the AMPKα agonist metformin, activation of AMPKα and subsequent activation of mTOR were not observed in SIRT4-deficient cells. Consistently, activation of AMPKα expression by metformin significantly eliminated the hyperproliferative phenotype in SIRT4-negative cells, a result that was consistent with the data *in vivo*. Therefore, SIRT4 deletion-dependent tumor biology is dependent mainly on activation of AMPKα stimulated by AMP/ADP. Metformin-activated AMPKα expression completely reversed the carcinogenic characteristics of SIRT4-negative cells. Interestingly, metformin had almost no effect on SIRT4-control HCC cells, which may explain the resistance of metformin-treated HCC patients (39). However, more clinical evidence is needed to ascertain if SIRT4 expression in human HCC can predict patient responses to metformin itself or in combination with sorafenib.

Zhi and colleagues used tissue microarrays to measure SIRT4 expression in 90 HCC samples. IHC analyses showed that SIRT4 expression was down-regulated significantly in tumor tissues compared with that in paracancerous tissues. In addition, total proteins and RNA were extracted from 16 pairs of fresh HCC tissues and matched paracancerous tissues, and revealed identical results (53).

SIRT4 expression in peritumoral tissue of HCC patients was negatively correlated with tumor size, pathology grade, T stage, and clinical stage. In addition, Kaplan–Meier analysis and logarithmic rank tests showed that SIRT4 expression in the tissue around the HCC was positively correlated with the survival rate of HCC patients. However, SIRT4 expression in tumor tissues was not related to the factors mentioned above. Therefore, those results suggested that SIRT4 may have a tumor-inhibitory role in pericarcinomatous tissues of HCC by inhibiting the development and migration of tumor cells and, thus, improving the prognosis of patients. Therefore, according to SIRT4 expression in the paracancerous tissues of HCC, all HCC cases were divided into two categories (high SIRT4 expression and low SIRT4 expression). Using IHC analyses and flow cytometry (FCM), two interesting phenomena were discovered: (i) increased infiltration of tumor-associated macrophages (TAMs); (ii) a higher proportion of M2/M1 macrophages in the tissue around the HCC in the group of low SIRT4 expression. Macrophage infiltration may be caused by several monocyte/macrophage chemokines. Increased expression of monocyte chemotactic protein-1 (MCP-1) was caused by down-regulation of SIRT4 expression in HCC, and it was the reason for the increase in TAM infiltration. Down-regulation of SIRT4 expression can activate the nuclear factor-kappa B (NF-κB) pathway, which leads to upregulation of *MCP-1* expression downstream (53). Furthermore, whether SIRT4 can catalyze the ADP ribosylation of mitochondrial NF-κB, and thereby increase nuclear translocation of p65, remains to be studied.

Another phenomenon observed was an increase in the proportion of M2/M1 macrophages in the tissues around HCC (53). Tao and colleagues reported that SIRT4 can reprogram endotoxin tolerance and promote acute inflammation of monocytes (54), thereby revealing the role of SIRT4 in immunoregulation. HCC-conditioned medium (HCM) was found to inhibit SIRT4 expression, and SIRT4 silencing stimulated macrophages to M2 polarization (53). The products of lipid metabolism play an important part in changing the function of macrophages and in inflammation (55).

M2 polarization of macrophages is related to activation of oxidative metabolism, including the functional TCA cycle promoted by glutamine and glucose catabolism, the increase of mitochondrial oxidative phosphorylation and fortification of fatty-acid oxidation (FAO) (56). SIRT4 is also involved in lipid metabolism and glutamine metabolism. It was shown that SIRT4 inhibits peroxisome proliferator-activated receptor α (PPARα) activity, thereby inhibiting FAO in the liver. More explicitly, SIRT4 disrupted the interaction of SIRT1 and PPARα, hence the activation of PPARα transcriptional activity by SIRT1 was inhibited (9, 57, 58).

In SIRT4-silenced TAMs, expression of FAO genes, including *MCAD*, *PDK4*, *CPT1*, *PGC1-α*, *PPARδ*, and *PPARα*, was increased, as was that of phosphorylated signal transducer and activator of transcription 3 (p-STAT3), which is necessary for differentiation into the M2 phenotype. Moreover, PPARδ suppressed by retroviruses reversed the effect of SIRT4-KO on expression of the phenotypic markers of TAMs. Therefore, SIRT4 silencing regulates TAMs to M2-like polarization through a FAO-PPARδ-STAT3 signaling pathway. Moreover, in the paracancerous tissues of HCC with low expression of SIRT4, the apoptosis rate of M1-like TAMs was high. Furthermore, the high expression of interleukin (IL)-10 induced by SIRT4 silencing in M2-like TAMs can prompt the apoptosis of M1 macrophages (53). Consistent with data from other studies, a high proportion of M2/M1 macrophages in pericarcinomatous tissue is associated with a poor prognosis in patients after tumor resection (59).

In HCM-stimulated TAMs, SIRT4 silencing: (i) promotes IL-10 expression but also promotes the production of cytokines, including IL-6 and vascular endothelial growth factor, which have been reported to prompt tumor progression; (ii) inhibits IL-12 production, enhances anti-tumor immunity, and prevents cancer development. Therefore, SIRT4 silencing in macrophages prominently prompts macrophage-induced tumor development *in vitro* and *in vivo* (53). The potential influence of SIRT4 on the STAT3-IL-6 axis needs to be studied further.

AMPK may also be a crucial moiety for FAO to regulate macrophages (60). AMPK can activate FAO at the expense of FA synthesis through at least two mechanisms: (i) phosphorylation and inactivation of ACC (the first step in FA synthesis); (ii) activation of PGC1-α which, in turn, stimulates the biogenesis and function of mitochondria. As mentioned above, SIRT4 can also affect the AMPK pathway, so whether the M2 polarization of TAMs is related to the SIRT4-AMPK-FAO axis merits further study.

Most tumor cells (including HCC cells) use aerobic glycolysis to maintain anabolic growth, and the preference for glycolysis often leads to adverse clinical outcomes. As mentioned above, SIRT4 can inhibit the PDHC to participate in aerobic glycolysis. HCC cells that overexpress SIRT4 show a reduced ability for glycolysis, and the mRNA and protein expression of key enzymes in glycolysis is also decreased (61).

Researchers have discovered that upregulation of the histone methyltransferase SET8 is positively correlated with poor survival in HCC patients, and aggravates glycolysis changes and tumor progression *in vitro* and *in vivo*. Notably, enhanced expression of SIRT4 is sufficient to reverse the increased expression of key glycolytic genes in SET8-overexpressed cells. The enhanced aerobic glycolysis observed in cells with SET8 overexpression is the result of SIRT4 inactivation (61). SET8 is the only known monomethyltransferase during monomethylation of histone H4 lysine 20 (H4K20me1) (62). Expression of SIRT4 mRNA and protein is decreased in HCC cells stably expressing SET8, whereas that of H4K20me1 is increased prominently. Chromatin immunoprecipitation (ChIP) studies have demonstrated that H4K20me1 is enriched in the SIRT4 promoter region. Those results suggest that SET8 inhibits the transcription of SIRT4 through H4K20me1. Krüppel-like factor 4 (KLF4) has a repressive role in the glycolysis of HCC cells, whereas mitochondrial oxygen consumption is enhanced. ChIP studies have shown that KLF4 binds directly to the SIRT4 promoter to aid its transcription. Also, SET8 and KLF4 are co-precipitated and co-localized, and SET8 overexpression leads to prominent reductions in expression of KLF4 mRNA and protein. However, the mechanism is not clear and may be related to DNA methylation (61).

Researchers have established a senescence model of the SMCC7721 liver-cancer cell line induced by doxorubicin. Use of the β-galactosidase method to measure cell senescence revealed that doxorubicin-induced SMCC7721 senescent cells had increased expression of the senescence-related proteins p53 and P16, increased expression of SIRT4, and blocked the Janus kinase 2 (JAK2) signaling pathway. In this model, SIRT4 overexpression could increase expression of the senescence-related protein p53, whereas the JAK2 signaling pathway was inhibited. CP-690550 (inhibitor of the JAK2/STAT3 signaling pathway) enhanced the cell senescence induced by doxorubicin significantly. Hence, SIRT4 promoted doxorubicin-induced senescence of SMCC7721 cells by inhibiting the JAK2/STAT3 signaling pathway (63).

**Figure 3:**
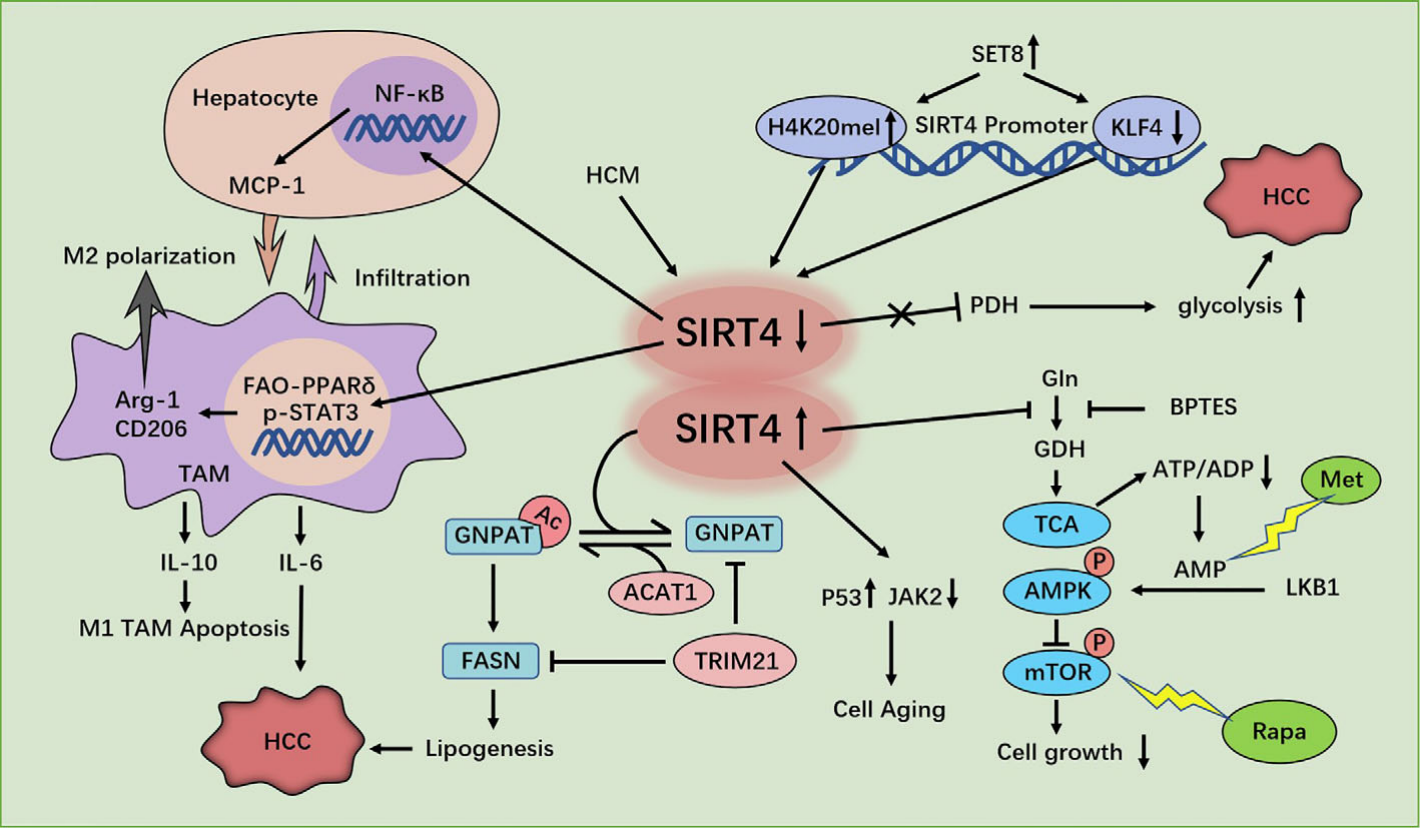
Regulation of HCC cells by SIRT4. SIRT4 depletion promotes hepatocellular carcinoma (HCC) genesis by regulating the AMPKα-mTOR axis. SIRT4 silencing in tumor-associated macrophages (TAMs) promotes HCC development through PPAR signaling-mediated macrophage activation. SET8 inhibits sirt4 transcription by regulating H4K20me1 and KLF4, thereby enhancing aerobic glycolysis and promoting the growth of HCC cells. SIRT4 deficiency promotes GNPAT acetylation, inhibits FASN degradation, promotes lipid synthesis, and promotes HCC. SIRT4 promotes the senescence of SMCC7721 cells in HCC by inhibiting the JAK2-STAT3 signaling pathway.

SIRT4 deacetylates glycerol O-acyltransferase (GNPAT) (64), expression of which is upregulated and which promotes FA synthesis in HCC cells at the K128 residue, while acetyl-CoA acetyltransferase 1 (ACAT1) (65), which is a mitochondrial enzyme, acetylates GNPAT at K128. Moreover, SIRT4 can antagonize ACAT1-mediated GNPAT acetylation. That is, SIRT4 deacetylates GNPAT at K128 with dissociation of ACAT1. In addition, ectopic expression of SIRT4 WT leads to a reduction in expression of GNPAT protein without a change in mRNA expression. This phenomenon occurs because deacetylation can promote TRIM21-mediated ubiquitination and degradation of GNPAT (66). Menendez and colleagues found that GNPAT interacts closely with fatty acid synthase (FASN) (67) which is a key enzyme in malonyl-CoA synthesis of long-chain FAs. FASN expression is up-regulated in many types of cancer, and is closely related to tumor invasiveness and a poor prognosis (68). GNPAT acetylation can inhibit TRIM21-mediated FASN degradation and promote lipid synthesis and tumor metabolism. In brief, SIRT4 deacetylates and reduces expression of GNPAT, which can promote lipid metabolism and hepatocarcinogenesis by stabilizing FASN (66) (**Figure 3**).

#### Pancreatic Cancer (PC)

In PC cell lines, SIRT4 overexpression strongly inhibits cell proliferation and increases apoptosis. It has been shown that SIRT4 is a negative regulator of aerobic glycolysis. SIRT4 mediates the effects of ubiquitin-like plant homologous domain and ring finger domain 1 (UHRF1) (69) on the proliferation and glycolysis metabolism in PC cells (70). Using an extracellular flux analyzer in seahorses to measure glycolysis, Hu and colleagues observed that SIRT4 overexpression inhibited the extracellular acidification rate, thereby indicating its negative regulation of aerobic glycolysis. In addition, if SIRT4 is overexpressed, the oxygen-consumption rate is increased accordingly, thereby supporting the positive role of SIRT4 in mitochondrial respiration (70). Conversely, UHRF1 (an epigenetic modifier (71), overexpressed in PC cells) predicts a poor prognosis and prompts PC-cell proliferation by promoting aerobic glycolysis, and positively regulates the transcriptional activity of HIF1 α in a dose-dependent manner. Whether using TCGA database, or employing IHC analyses of 70 PC patients, or experimenting with PC cell lines, a negative correlation between UHRF1 and SIRT4 has been confirmed. ChIP analyses have shown that UHRF1 combines specifically with the SIRT4 promoter to inhibit SIRT4 expression in PC cells. SIRT4 overexpression reduces the protein expression of HIF1α, as well as the expression of glycolysis genes (*GLUT1*, *HK2*, and *LDHA*) at mRNA and protein levels. *In vitro* studies have also shown a significant negative correlation between SIRT4 and glycolysis genes (*GLUT1*, *HK2* and *LDHA*) in PC patients in a cohort in TCGA database (70). In brief, UHRF1 promotes aerobic glycolysis and proliferation of PC cells *via* suppression of SIRT4 expression.

**Figure 4:**
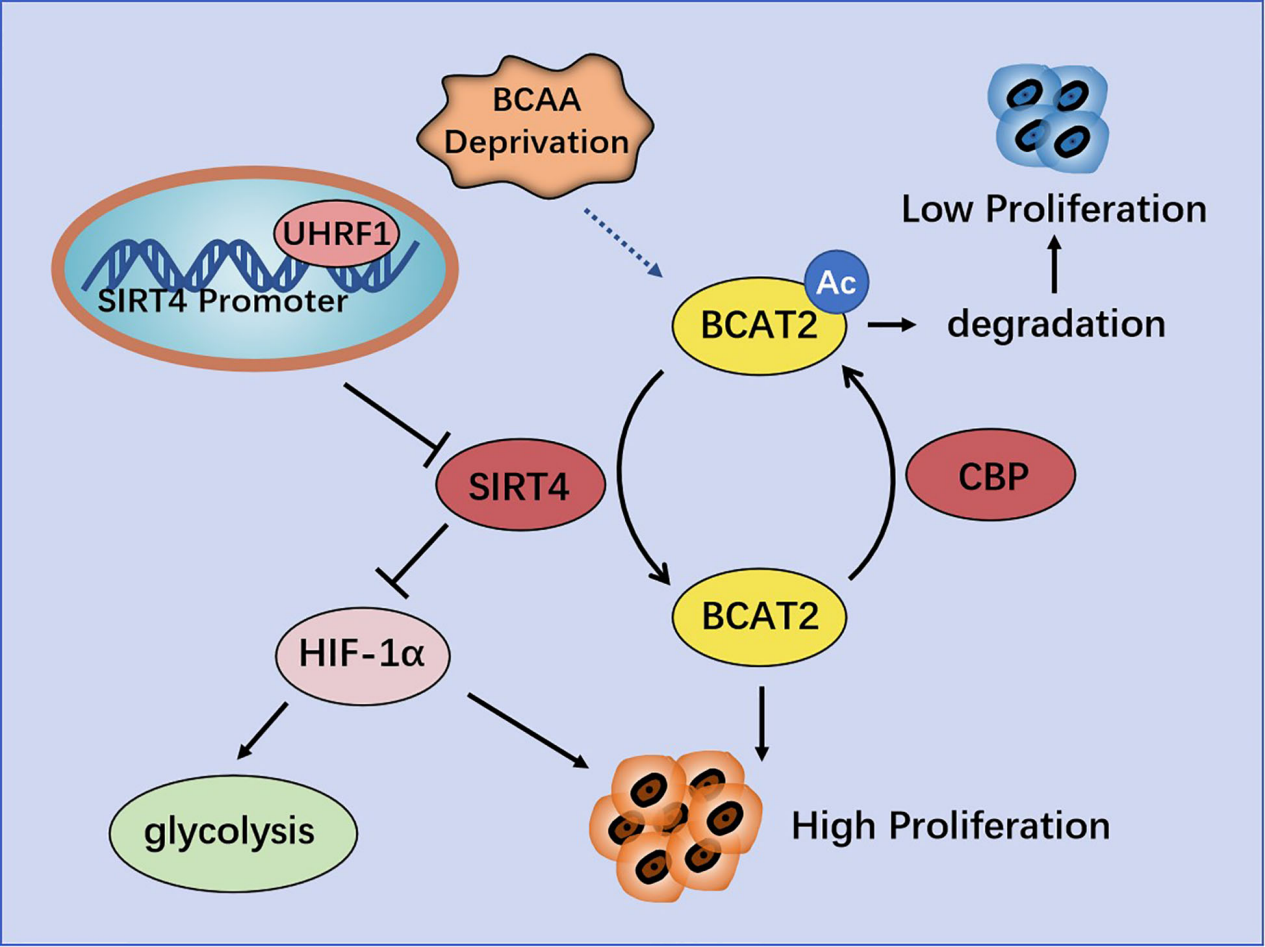
Regulation of pancreatic cancer (PC) by SIRT4. UHRF1 promotes aerobic glycolysis and proliferation of PC cells by inhibiting SIRT4 expression. BCAT2 is deacetylated and stabilized by SIRT4, and is acetylated and degenerated by CBP.

However, Lei and colleagues claimed that SIRT4 is involved in catabolism of branched-chain amino acids and promotes cell proliferation and tumor growth in PDAC by regulating BCAT2. They found that BCAT2 could be deacetylated and stabilized by SIRT4 at K44. Conversely, cAMP-responsive element-binding (CREB)-binding protein (CBP) is the acetyltransferase of BCAT2. BCAT2 acetylation leads to its degradation through a ubiquitin-proteasome pathway, and is stimulated by deprivation of branched-chain amino acids (34) (**Figure 4**).

#### Gastric and Esophageal Cancer

In a study of 75 patients with gastric adenocarcinoma in 2015, SIRT4 expression in tumor tissue was significantly lower than that in corresponding normal tissues. In addition, SIRT4 expression was negatively correlated with the pathologic grade, depth of tumor invasion, number of positive lymph nodes, and International Union Against Cancer stage (72).

Sun and colleagues collected 86 pairs of GC tissues and paracancerous normal tissues. They also found that SIRT4 expression was down-regulated in GC tissues and negatively correlated with tumor size, pathologic grade, and lymph-node metastasis. Moreover, the survival rate of patients with low expression of SIRT4 was decreased (73).

In GC cell lines, SIRT4 expression is decreased, and functional experiments have shown that SIRT4 inhibits the proliferation, migration, and invasion of GC cells. Enzyme-linked immunosorbent assays have shown that ectopic expression of SIRT4 inhibits secretion of matrix metallopeptidase (MMP)2 and MMP9 (73), which are closely related to tumor invasiveness in humans and a poor prognosis (74). SIRT4 regulates epithelial–mesenchymal transition (EMT) to control the migration and invasion of cells. SIRT4 overexpression significantly upregulates expression of the mRNA and protein of E-cadherin, down-regulates expression of the protein of N-cadherin and vimentin, but has no effect on their mRNA. It has been postulated that SIRT4 may inhibit EMT by regulating E-cadherin expression. Measurement of the RNA expression of EMT-related transcription factors (slug, snail, and twist) has revealed that ectopic expression of SIRT4 reduces expression of slug but has no effect on snail or twist (73).

Researchers have shown that SIRT4 overexpression induced arrest of the G1 phase of the cell cycle by inhibiting expression of phosphorylated extracellular signal-regulated kinase (p-ERK), cyclin D and cyclin E (75). FCM and staining (propidium iodide) revealed that SIRT4 overexpression prominently increased the proportion of cells in the G1 phase and decreased the number of cells in the S phase of the cell cycle. SIRT4 inhibited expression of cyclin D and cyclin E prominently. In addition, SIRT4 predominantly decreased p-ERK expression, but did not affect ERK expression (75). It has been reported that ERK controls the G1 phase of the cell cycle by regulating expression of cyclin D (76). In contrast, SIRT4 overexpression does not affect the apoptosis of GC cells (75).

Nakahara and co-workers evaluated 172 patients with esophageal squamous cell carcinoma (ESCC) who underwent resection. They measured expression of SIRT4 mRNA in cohort 1 (n = 79) and measured expression of SIRT4 protein in cohort 2 (n = 93). They found that SIRT4 expression was not correlated with any clinicopathologic parameter in either cohort. However, in cohort 1, the overall survival rate of patients with low expression of SIRT4 was lower than that of cases with high SIRT4 expression. In cohort 2, the rate of overall survival and disease-free survival of SIRT4-negative patients was lower than that of their SIRT4-positive counterparts. Multivariate analysis showed that SIRT4 expression was an independent prognostic factor for overall survival. Also, the rate of distant recurrence of SIRT4-negative patients was notably higher than that of SIRT4-positive cases. Moreover, SIRT4-KO can predominantly increase GDH activity and promote the proliferation and migration of esophageal-cancer cells *in vitro* (77). Recently, our team witnessed that SIRT4 is a specific target gene for micro (mi)R-424-5p in ESCC and negatively modulated by miR-424-5p. miR-424-5p expression was up-regulated in ESCC and dramatically facilitated the proliferative and migratory capacity of ESCC cells. SIRT4 overexpression strongly rescued the promotive influence of miR-424-5p on the proliferative and migratory capacity of ESCC cells. Hence, manipulation of the miR-424-5p/SIRT4 axis could provide a novel strategy for further ESCC treatment (78).

#### Colorectal Cancer (CRC)

Miyo and colleagues collected 142 samples of CRC tissue and part of its paracancerous tissue, as well as 38 samples of colorectal adenomas tissue, from patients after resection. They found that the proportion of patients with high expression of SIRT4 in normal tissues (61.1%) was greater than that for patients with adenomas (52.6%) or CRC (52.5%). SIRT4 expression was negatively correlated with tumor–node–metastasis (TNM) stage, but not with sex, age, venous invasion or histology type. The recurrence rate of patients with low expression of SIRT4 was increased, and 5-year overall survival rate tended to decrease. It was also found that SIRT4 inhibited the proliferation, invasion, and migration of CRC cell lines. SIRT4 inhibited the enzyme activity of GDH, and E-cadherin gene (CDH1) expression was up-regulated notably by SIRT4. Immunofluorescence staining also demonstrated that SIRT4 overexpression could increase E-cadherin expression. In addition, it was shown that vimentin expression was predominantly down-regulated by SIRT4 overexpression (79). Shen and colleagues showed that E-cadherin expression was inhibited by the zinc finger E-box binding homeobox (ZEB) family, whereas expression of the ZEB family was inhibited by miRNA-200c (80). MiRNA-200c expression was inhibited by SIRT4, but SIRT4 had an inconsistent effect on ZEB1 expression, suggesting that the mechanism by which SIRT4 regulates E-cadherin expression may be different. Further studies showed that inhibition of glutamine metabolism by SIRT4 led to positive regulation of E-cadherin expression. In addition, α-ketoglutarate (α-KG) eliminated the induction of E-cadherin expression by SIRT4, which suggested that SIRT4 inhibited EMT by inactivating GDH and reducing a-KG expression in cells. Those data revealed other molecular mechanisms of glutamine-metabolism regulation, which affected E-cadherin expression through SIRT4 and, in turn, affected cell mobility. In brief, glutamine metabolism may be important for the mobility of cancer cells (79).

**Figure 5:**
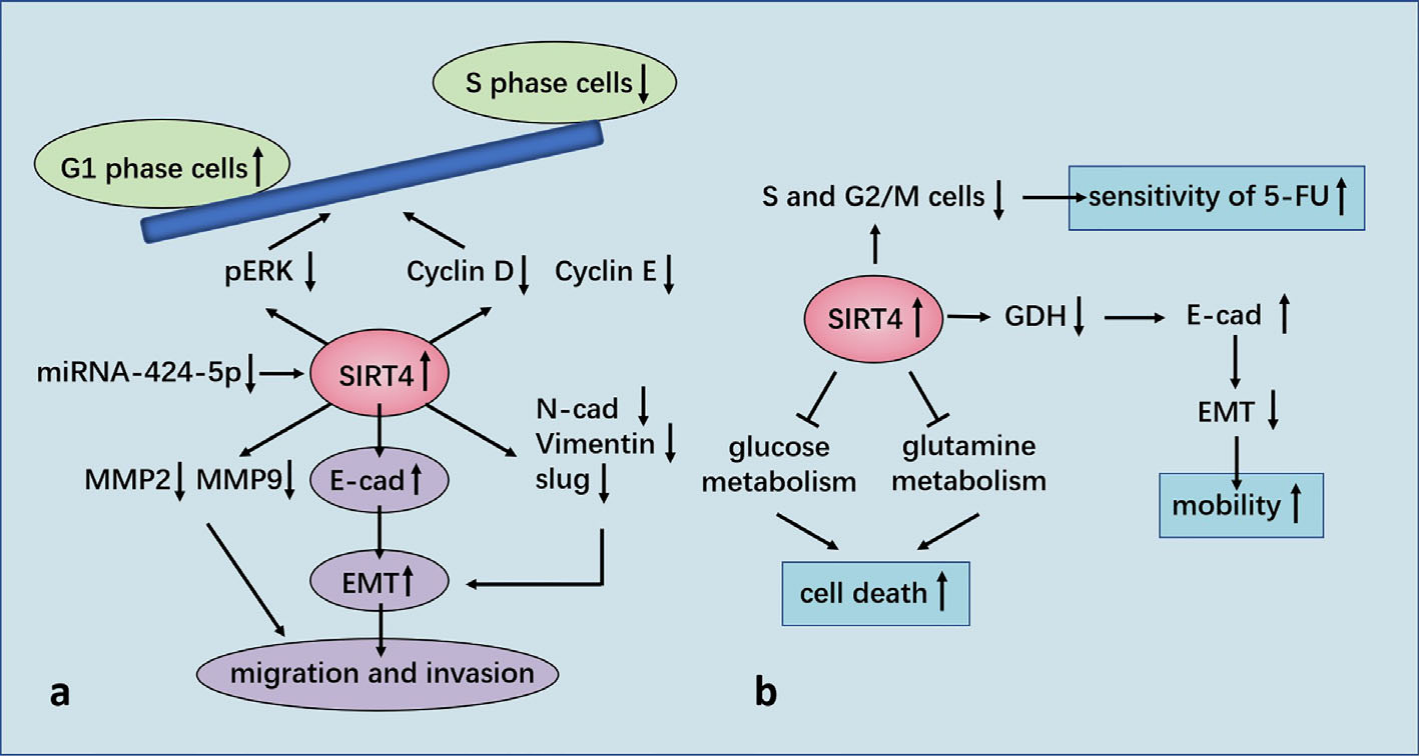
**(A)** Regulation of GC by SIRT4. SIRT4 inhibits the proliferation, migration, and invasion of GC cells by regulating epithelial–mesenchymal transition (EMT). SIRT4 overexpression induces stagnation of the G1 phase of the cell cycle by inhibiting expression of p-ERK, cyclin D, and cyclin E. SIRT4 is a specific target gene for miR-424-5p in ESCC and negatively modulated by miR-424-5p. **(B)** Regulation of CRC by SIRT4. SIRT4 expression leads to positive regulation of E-cadherin expression by inhibition of glutamate dehydrogenase (GDH) expression, which inhibits EMT and influences cell mobility. SIRT4 inhibits glutamine metabolism and induces death of colorectral cancer (CRC) cells in conjunction with glycolysis inhibition. SIRT4 increases the sensitivity of CRC cells to 5-FU by delaying mitosis.

Huang and colleagues used tissue microarrays in 89 cases of CRC. They found that SIRT4 expression in CRC tissues was notably lower than that in normal tissues. The lower the SIRT4 expression, the worse was the pathologic differentiation and the lower was the overall survival rate. mRNA expression of SIRT2, SIRT4 and SIRT5 in 16 pairs of CRC tissues and adjacent normal tissues showed that SIRT4 expression was reduced significantly, whereas that of SIRT2 and SIRT5 showed no significant changes. Analyses of 236 cases of CRC samples and 22 normal colorectal-tissue samples from TCGA database provided data that were consistent with the results stated above. Moreover, SIRT4 expression was down-regulated in the early stage and, importantly, its low expression was maintained during cancer progression, suggesting that down-regulation of SIRT4 expression may be necessary for tumorigenesis and maintenance of tumor cells. Further studies showed that SIRT4 overexpression inhibited proliferation of CRC cells *in vivo* and *in vitro*. SIRT4 inhibited glutamine metabolism and cooperated with glycolysis inhibition to induce the death of CRC cells. In addition, SIRT4 overexpression significantly reduced the rates of the S phase and G2/M phases of two CRC cell lines after 5-fluorouracil treatment, but had no effect on the apoptosis rate, indicating that SIRT4 increased the sensitivity of CRC cells to 5-fluorouracil by delaying mitosis (81) (**Figure 5**).

### Urogenital Neoplasms

#### Breast Cancer

High-throughput real-time polymerase chain reaction analyses have shown that SIRT4 expression in breast-cancer tissue is predominantly lower than that in paracancerous tissue (82). The analysis of TCGA and 3 Gene Expression Omnibus (GEO) datasets showed that decreased SIRT4 expression correlated with increased risk of disease progression and poor clinical outcome. Besides, immunoblotting and IHC staining also showed a significant decrease in SIRT4 protein levels in breast tumor tissues (83). Wang and colleagues found that down-regulation of SIRT4 expression promoted glutamine decomposition, promoted the growth of breast-cancer cells and inhibited their apoptosis. ChIP analyses demonstrated that C-terminal-binding protein (CtBP) was bound to the *SIRT4* promoter and inhibited its expression directly. Inhibition of *SIRT4* expression by CtBP helped to maintain the pH homeostasis of breast-cancer cells and was beneficial to their growth. CtBP also down-regulated *SIRT4* expression to promote the growth of breast-cancer cells and inhibited their apoptosis (84).

Glucose and glutamine are essential components for cell growth. The maximum yield from the original nutrients can be achieved by coordinating the metabolic pathways of different substrates. A mechanism for coordinating consumption of glucose and glutamine in cancer cells has been proposed: the CtBP-SIRT4-GDH axis mediates crosstalk between glycolysis and glutamine decomposition. Synergistic consumption of glucose and glutamine among different cell lines was observed. That is, glutamine consumption was delayed under low and high levels of glucose. Application of glycolysis inhibitors showed that, with an increase in the drug dose, the decrease in glutamine consumption was consistent with a decrease in glucose consumption (85).

Researchers found that when cells were cultured in low-glucose medium, SIRT4 expression increased in a time-dependent manner. Application of a glycolysis inhibitor showed that restricted glycolysis upregulated SIRT4 expression and eroded GDH activity. Accordingly, treatment with a glycolysis inhibitor also up-regulated p21 expression, indicating that cell growth stopped, which was similar to the effect of DNA damage. This synergistic effect was related to the inhibition of SIRT4 expression by CtBP at high glucose levels, and GDH activity also indicated that CtBP needed a high glucose supply to inhibit *SIRT4* expression. However, low glucose abolished the binding of CtBP to the SIRT4 promoter and inhibition of SIRT4 expression. Dimerized CtBP has great potential to inhibit expression of target genes. Use of the bimolecular fluorescence complementation method revealed that a low glucose supply reduced the binding activity of CtBP as a co-inhibitor by inhibiting CtBP dimerization (85).

Xing and collaborators showed that SIRT4 increased the sensitivity of breast-cancer cells to tamoxifen. SIRT4 overexpression decreased the half-maximal inhibitory concentration of tamoxifen in estrogen receptor (ER)-negative MCF7 and T47D cells, whereas its depletion increased it. Glutamine is an essential nutrient for the growth and survival of cells. Tamoxifen inhibited the proliferation of ER-negative cells by inhibiting glutamine uptake. Cells treated with tamoxifen after SIRT4 overexpression consumed less glutamine than cells treated with tamoxifen alone. This observation suggested that SIRT4 and tamoxifen work together to reduce glutamine absorption by ER-positive breast-cancer cells. In addition, SIRT4 enhanced inhibition of the proliferation and apoptosis of ER-positive breast-cancer cells induced by tamoxifen (86).

STAT3 is activated in breast cancer, and increased phosphorylation of the tyrosine 705 (Y705) site in STAT3 has been observed in tamoxifen-resistant MCF7/TAM cells (87). STAT3 mediates expression of many genes in response to cellular stimuli, and overactivation of STAT3 is led by phosphorylation of the Y705 site (88). Importantly, SIRT4 inhibits the phosphorylation and nuclear translocation of STAT3, thereby inhibiting the STAT3 signaling pathway, and amplification of the target genes *MYC* and *CCND1* of the STAT3 signaling pathway provide tamoxifen resistance in ER-positive breast-cancer cells (89, 90). The transcription and translation of *MYC* and *CCND1* are also blocked by SIRT4. In addition, activation or inhibition of STAT3 expression can counteract the effect of SIRT4 overexpression/depletion on cell proliferation. STAT3 signal transduction is stimulated by cytokines such as IL-6. If SIRT4 is overexpressed, IL-6 release in ER-positive breast-cancer cells decrease, and inhibition of STAT3 expression induced by SIRT4 is reversed after IL-6 treatment. Those data suggest that SIRT4 enhances the sensitivity of ER-positive breast-cancer cells to tamoxifen by inhibiting the IL-6/STAT3 pathway (86).

**Figure 6:**
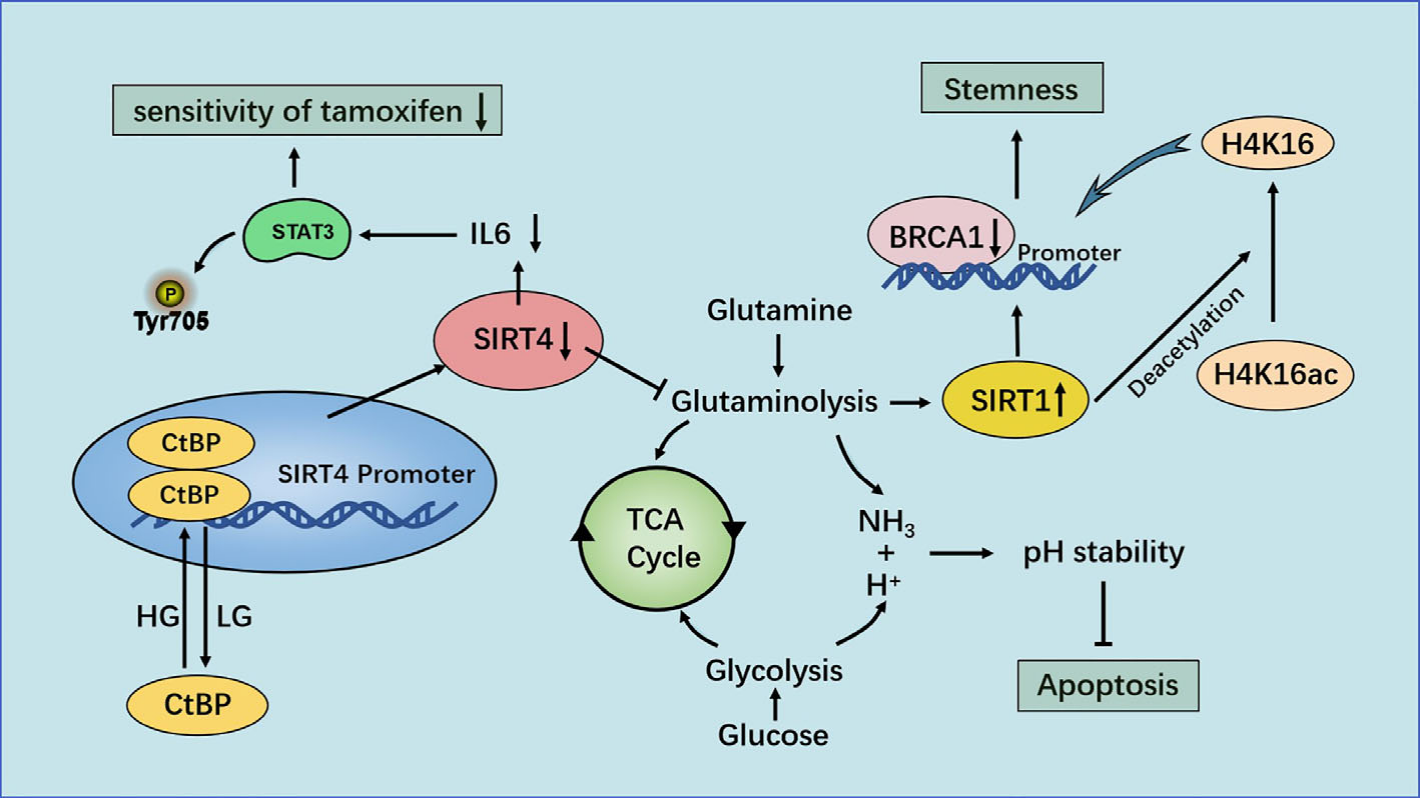
Regulation of breast cancer by SIRT4. Under the condition of high glucose levels, CtBP dimerization helps CtBP to promote glutamine metabolism by inhibiting SIRT4 expression. This action can help maintain the pH homeostasis of cells, promote the growth of breast-cancer cells and inhibit their apoptosis. SIRT4 enhances the sensitivity of ER-positive breast cancer cells to tamoxifen by inhibiting glutamine metabolism and the IL-6-STAT3 signaling pathway.

Recent studies have shown that SIRT4 plays a novel part in the negative regulation of mammary-gland development and stemness. SIRT4 can inhibit the self-renewal and expansion of breast tumor-initiating cells and may protect mice from breast tumors and lung metastasis by preventing the formation of mammary stem cells. In this mechanism, SIRT4 negatively regulates SIRT1 expression by inhibiting glutamine metabolism, ensuring inhibition of breast stem cells. H4K16ac and breast cancer susceptibility gene 1 (BRCA1) are new major targets for SIRT4 in breast cancer. SIRT4 deficiency increases the expression of acetyl-histone H3 at lys9 (H3K9ac), acetyl-histone H4 at lys16 (H4K16ac) and stem-cell markers, and down-regulates BRCA1 expression by regulating H4K16ac rather than well-defined H3K9ac. These processes cannot be separated from SIRT1, which regulates the acetylation pattern of histone H4 and regulates stemness through BRCA1 (83) (**Figure 6**).

#### Ovarian Cancer

Use of an online database of Kaplan–Meier plots ascertained the prognostic value of expression of SIRT4 mRNA in patients with ovarian cancer. High expression of SIRT4 mRNA indicated the poor survival of patients with ovarian cancer. In pathologic grade-III carcinoma and all clinical stages, overexpression of SIRT4 mRNA was correlated with poor overall survival. In addition, SIRT4 expression was negatively correlated with the overall survival of patients treated with drugs (91).

#### Endometrioid Adenocarcinoma

IHC analyses were undertaken to measure expression of SIRT4 protein in 65 samples of endometrioid-adenocarcinoma tissue using microarrays. SIRT4 expression was significantly lower in endometrioid-adenocarcinoma tissue than that in non-neoplastic tissue. In addition, SIRT4 expression was lower in tissues from patients with an advanced stage of endometrioid-adenocarcinoma according to American Joint Committee on Cancer classification (92).

#### Prostate Cancer

Li and colleagues found that, based on IHC analyses, SIRT4 expression in prostate-cancer tissues was significantly lower than that in matched paracancerous tissues, and that expression of SIRT4 protein was low in 9/12 frozen prostate-cancer tissues. They discovered that the P21 activated kinase 6-SIRT4-adenine nucleotide translocase-2 (PAK6-SIRT4-ANT2) complex could inhibit the apoptosis of prostate-cancer cells (93). Previously, it had been reported that ANT2 (94), in the inner membrane of mitochondria, is the regulatory substrate of SIRT4. SIRT4 can regulate expression of ANT2 protein through deacetylation, and maintain the energy balance in mitochondria by coupling with ANT2 (18, 95). Furthermore, they found that SIRT4 regulates the acetylation level of ANT2 on the K105 site and degrades ANT2 by promoting its ubiquitination, which is dependent upon the deacetylation activity of SIRT4. Notably, regulation of acetylation activity of ANT2 by SIRT4 was dependent upon the presence of glutamine in the medium (93). In addition, P21 activated kinase 6 (PAK6) (96) is a conserved family of serine/threonine kinases, which is also located mainly in the mitochondrial inner membrane. PAK6 was found to promote SIRT4 ubiquitin-mediated proteolysis. Moreover, PAK6 phosphorylated ANT2 directly at the T107 site, and directly/indirectly affected ANT2 acetylation through SIRT4. The interaction between phosphorylation and deacetylation of ANT2 regulated the apoptosis of prostate-cancer cells. In summary, multiple post-translational modifications between proteins in the PAK6-SIRT4-ANT2 complex (including the promotion of ubiquitin degradation through deacetylation and the interaction between acetylation at K105 and phosphorylation at T107) lead to various modifications that interact with the target protein ANT2. These modifications, in turn, eventually regulate the apoptosis of prostate-cancer cells, and lead to tumor growth in the body (93).

#### Clear Cell Renal Cell Carcinoma (ccRCC)

Using publicly available TCGA pan-cancer datasets, as well as western blotting and real-time reverse transcription-quantitative polymerase chain reaction, the expression and prognostic value of SIRTs in ccRCC was evaluated. SIRT4 expression in ccRCC was lower than that in normal tissues, and low SIRT4 expression was obviously involved with poor overall survival and advanced tumor stage in ccRCC patients (97, 98). Wang and coworkers also indicated that SIRT4 overexpression significantly reduced the proliferation, migration and invasion ability of ccRCC cells *in vitro* (98).

#### Bladder Cancer

Consistent with the findings in ESCC, Yu and colleagues found that miR-424 could regulate SIRT4 expression in bladder cancer. They found that high expression of miR-424 was closely related to the poor clinical prognosis of patients with bladder cancer. In this mechanism, cisplatin induced miR-424 expression in bladder cancer cells in a HIF-1 α-dependent manner, and miR-424 conferred cisplatin resistance on bladder cancer cells *in vitro* and *in vivo*. Importantly, miR-424 mediated this resistance by down-regulation of expression of SIRT4 and pro-apoptotic UNC5B. SIRT4 knockout prohibited cisplatin-mediated PARP proteolysis and reduced the sensitivity of these cells to cisplatin (99).

### Respiratory Neoplasms

#### Lung Neoplasms

Low expression of SIRT4 in non-small cell lung cancer (NSCLC) patients is related to clinicopathologic factors and a poor prognosis. Carrying out IHC analyses on 133 lung-cancer tissues, it was found that SIRT4 expression was decreased dramatically in 52.6% of lung-cancer tissues. There was no significant difference between SIRT4 status and age, sex or histology differentiation, but it was negatively correlated with TNM stage. In addition, the proportion of SIRT4 down-regulation in adenocarcinoma (52/84, 61.9%) was higher than that in squamous cell carcinoma (18/49, 36.73%). Patients with low expression of SIRT4 showed stronger expression of Ki-67 and shorter survival than those with high SIRT4 expression (41).

Upregulation of SIRT4 expression inhibited the proliferation, invasion and migration of lung-cancer cell lines. FCM showed that the percentage of cells in the S phase decreased in cell lines with SIRT4 transfection, suggesting that SIRT4 may inhibit cell growth by inhibiting the transition from the S phase to the G1 phase in mice. SIRT4 damaged mitochondrial fission and inhibited the activity of the mitochondrial fission-related protein Drp1. Mitochondria in cells overexpressing SIRT4 found it easier to fuse and had longer shapes. SIRT4 overexpression resulted in a significant down-regulation of Drp1 expression (41). The active form of Drp1, phosphate Drp1 (S616), promoted the entrapment of Drp1 around the mitochondrial membrane and induced an increase in mitochondrial fission (100). SIRT4 attenuated mitochondrial fission by inhibiting Drp1S616 phosphorylation. SIRT4 inhibited the migration and invasion of lung-cancer cells by inhibiting the mitochondrial fission induced by Drp1. SIRT4 overexpression inhibited the activity of mitogen-activated protein kinase (MEK) and ERK significantly, whereas inhibition of MEK/ERK expression reversed the upregulation of p-Drp1 expression and enhanced the invasive ability and mitochondrial fission induced by SIRT4 depletion.

To evaluate further the function of SIRT4 in lung-cancer metastasis, 60 samples of primary NSCLC tissues and their corresponding lymph-node metastatic tumors were analyzed. SIRT4 expression in the latter was lower than that in corresponding primary NSCLC tissues. Also, there was a negative correlation between expression of SIRT4 and Drp1 in primary NSCLC tissues and lymph-node metastatic tumors. *In vivo* experiments also showed that SIRT4 overexpression delayed tumor growth notably, and that SIRT4 expression was negatively correlated with p-Drp1 expression in lung-cancer samples (41).

### Neoplasms of Circulatory Systems

#### Burkitt’s Lymphoma

In Myc-driven human Burkitt’s lymphoma cells, SIRT4 overexpression inhibits mitochondrial glutamine metabolism which, in turn, inhibits cell proliferation (101). Glutaminase is the first enzyme in the mitochondrial metabolism of glutamine, and its inhibition limits the entry of glutamine into the TCA cycle (102). It has been shown that if the glutaminase level is reduced using short-hairpin RNAs, SIRT4 overexpression no longer inhibits glutamine uptake (42). BPTES (glutaminase inhibitor) treatment can inhibit cell proliferation much more than that observed with SIRT4 overexpression, but the precise relationship between SIRT4 and glutaminase is not clear. The synergistic effect of SIRT4 and glycolysis inhibition leads to the death of Burkitt’s lymphoma cells. However, SIRT4 can regulate glutamine metabolism in cells independent of Myc. SIRT4 expression in Burkitt’s lymphoma cells does not affect expression of Myc and its target genes, which is incompatible with the effects seen in HCC cells and PC cells. This phenomenon may be caused by specificity to tumor tissues or organ functions. In a mouse model, SIRT4 deletion accelerated the formation of lymphoma and mortality in Em-Myc transgenic mice (101).

#### Acute Myeloid Leukemia

Bradbury and colleagues characterized expression of all 18 members of the HDAC family in primary acute myeloid leukemia blasts, and in four types of control cell (CD34+ progenitors from the umbilical cord, either quiescent or cycling (post-culture), cycling CD34+ progenitors from granulocyte-colony stimulating factor-stimulated adult donors, and peripheral blood mononuclear cells). Among them, SIRT4 expression was decreased in acute myeloid leukemia cells compared with that in control cells (103).

### Other Types of Neoplasms

#### Thyroid Cancer

Analyses of tissue samples from 89 patients with thyroid cancer by Chen and colleagues showed that expression of SIRT4 protein decreased significantly compared with that in normal tissue, but there was no significant correlation with clinicopathologic features. *In vitro* experiments showed that SIRT4 inhibited the proliferation, migration, and invasion of thyroid-cancer cells. SIRT4 overexpression blocked the G0/G1 phase of the cell cycle, and induced apoptosis. SIRT4 inhibited the migration and invasion of thyroid-cancer cells by inhibiting glutamine metabolism (104).

#### Head and Neck Squamous Cell Carcinoma (HNSCC)

Expression of mitochondrial tumor-suppressor genes (*SIRT3*, *SIRT4*, and *MTUS1*), a mitochondrial DNA repair gene (OGG1) and a proliferation marker (Ki-67) was measured by reverse transcription-quantitative polymerase chain reaction and real-time fluorescence quantitative polymerase chain reaction in HNSCC patients and controls by Mahjabeen and colleagues. Compared with the control group, expression of *SIRT3*, *SIRT4*, *MTUS1*, and OGG1 in HNSCC was down-regulated significantly, whereas Ki-67 expression was up-regulated. SIRT4 expression in the advanced clinical stage (III–IV) was significantly lower than that in early clinical stage (I–II), and SIRT4 expression was negatively correlated with TNM stage. A positive correlation between expression of SIRT4 and SIRT3, SIRT4 and OGG1 was observed (105). In conclusion, down-regulation of expression of certain mitochondrial tumor-suppressor genes, such as *SIRT3*, *SIRT4*, and *MTUS1*, in HNSCC cells indicates aggressive tumor behaviors, and may predict an unfavorable clinical outcome.

#### Neuroblastoma

Analyses of human neuroblastoma tissues and adjacent normal tissues were undertaken by Wang and colleagues. They showed that SIRT4 expression was decreased dramatically in neuroblastoma tissues. SIRT4 expression was negatively correlated with the stage (according to the International Neuroblastoma Staging System) and lymph-node metastasis. The duration of survival of neuroblastoma patients with low expression of SIRT4 was shortened significantly. *In vitro* experiments showed that SIRT4 overexpression reduced the proliferation, invasion, and migration of neuroblastoma cells significantly. Up-regulation of SIRT4 expression decreased the basic oxygen consumption rate of SH-SY5Y cells significantly, suggesting that up-regulation of SIRT4 expression inhibited respiration and decreased energy production in mitochondria. Simultaneously, up-regulation of SIRT4 expression diminished the value of spare respiratory capacity dramatically, indicating that up-regulation of SIRT4 expression may reduce maximum energy production in mitochondria and increase mitochondrial damage. SIRT4 overexpression reduced expression of SIRT1 protein (106). The role of SIRT1 in regulating tumors through energy metabolism has been demonstrated by several scholars. For example, SIRT1 was shown by Rodgers and colleagues to promote tumor development by interfering with mitochondrial biosynthesis (107). SIRT1 also interferes with glycolysis in tumor cells by activating HIF-2α, thereby regulating energy metabolism in tumors (108). In brief, SIRT4 exhibited a tumor-suppressor function in human neuroblastoma, and reduced the energy metabolism of tumor cells by inhibiting mitochondrial metabolism and SIRT1 expression (106) (**Table 1**).

**Table 1:** SIRT4 in various types of tumor.

Type of Tumor		SIRT4 Expression	Relationship with Clinicopathological Features	Relationship with Prognosis	Phenotype	Molecular Mechanism	Ref.
**Digestive System Neoplasm**	**HCC**	decrease	SIRT4 expression in the peripheral tissues of HCC tumors was negatively correlated with tumor size, pathological grading, stage T, and clinical stage.	Low levels of SIRT4 are associated with a shorter recurrence time. SIRT4 in the sparacancerous tissues were positively correlated with survival of HCC patients.	SIRT4 depletion promoted cell proliferation and migration in vitro, and promote tumorigenesis and lung metastasis *in vivo*. SIRT4 promotes aging of cells.	**Figure 3**	(39, 53, 61, 63, 66)
	**PC**	decrease	–	–	SIRT4 inhibits cell proliferation.	**Figure 4**	(34, 70),
	**GC and ESCC**	decrease	SIRT4 level was negatively correlated with tumor size, pathological grading, tumor invasion depth, number of positive lymph nodes, and UICC stage.	Survival rate of low expression of SIRT4 was decreased.	SIRT4 inhibits cell proliferation, migration, and invasion.	**Figure 5**	(72, 73, 75, 77, 78)
	**CRC**	decrease	SIRT4 expression was negatively correlated with TNM stage, but not with sex, age, venous invasion, or histological type.	The recurrence rate of patients with low expression of SIRT4 is increased, and the 5-year OS rate tended to decrease.	SIRT4 inhibits cell proliferation, migration, and invasion. SIRT4 increases the sensitivity of colorectal cancer cells to 5-FU.		(79, 81),
**Urogenital Neoplasms**	**Breast Cancer**	decrease	–	Loss of SIRT4 expression is strongly associated with short-term metastasis in breast cancer patients.	SIRT4 inhibits the growth of cells and increases the sensitivity of ER positive breast cancer to tamoxifen.	**Figure 6**	(82–86)
	**OC**	decrease	–	High SIRT4 levels suggest poor survival.	–	–	(91)
	**EA**	decrease	SIRT4 was lower in advanced AJCC stages.	–	–	–	(92)
	**Prostate Cancer**	decrease	–	–	SIRT4 inhibits the growth of cells.	PAK6-SIRT4-ANT2 complex regulates apoptosis of prostate cancer cells	(93)
	**ccRCC**	decrease	–	High SIRT4 levels suggest good prognosis.	overexpression of SIRT4 significantly reduced the proliferation, migration and invasion ability of ccRCC cells	–	(97, 98)
	**Bladder cancer**	–	–	–	–	miR-424 mediates CDDP resistance by down-regulation of SIRT4	(99)
**Respiratory Neoplasms**	**Lung Cancer**	decrease	SIRT4 negatively correlated with TNM. SIRT4 was lower in adenocarcinoma than in squamous cell carcinoma. Low SIRT4 expression showed stronger Ki-67 expression.	The survival rate of SIRT4 negative patients is significantly lower than positive.	SIRT4 inhibits cell proliferation, invasion, and migration. SIRT4 significantly delayed tumor growth *in vivo*.	SIRT4 inhibits malignancy progression of NSCLCs through mitochondrial dynamics mediated by the ERK-Drp1 pathway.	(41)
**Circulatory Tumor**	**Burkitt Lymphoma**	–	–	–	SIRT4 inhibits cell proliferation. SIRT4 deficiency accelerated lymphoma formation and mortality in Em-Myc transgenic mice.	SIRT4 inhibits cell proliferation by inhibiting mitochondrial glutamine metabolism.	(101)
	**AML**	decrease	–	–	–	–	(103)
**Other Tumor Types**	**Thyroid Cancer**	decrease	SIRT4 were independent of age, tumor size, TNM or AJCC.	–	SIRT4 inhibits cell proliferation, invasion, and migration.	SIRT4 blocks the cell cycle and induced apoptosis.SIRT4 inhibited BCPAP migration and invasion by inhibiting glutamine metabolism.	(104)
	**HNSCC**	decrease	SIRT4 in advance clinical stage (III-IV) was lower than in early stage (I-II). SIRT4 was negatively correlated with TNM.	–	–	–	(105)
	**NB**	decrease	SIRT4 expression was negatively correlated with INSS and lymph node metastasis.	The survival time of NB patients with low expression of SIRT4 was significantly shortened.	SIRT4 inhibits cell proliferation, invasion, and migration.	SIRT4 inhibits mitochondrial metabolism and SIRT1 expression to reduce tumor energy metabolism.	(106)

## Conclusions

Substantial studies have focused on SIRT4 from structure to location, and from enzyme activity to function. Structure determines the localization and enzyme activity, which determines function. SIRT4 with a N-terminal MTS is responsible for localization in the mitochondrial cavity. Recent studies have demonstrated that SIRT4 is also present in the nucleus and cytoplasm, which may involve new crosstalk. SIRT4 has various enzyme activities. SIRT5 has distinct desuccinylase activity, and SIRT1–3 have remarkable deacetylase activity (109). The main enzyme activity of SIRT4 is not known because the identity of its specific substrates is not known.

However, various physiologic functions regulated by SIRT4 have been identified. SIRT4 has important roles in energy metabolism and oxidative stress, metabolic diseases and cancer, physical fitness, health, and ageing (110).

We reviewed mainly the function of SIRT4 in various malignant neoplasms. SIRT4 expression is down-regulated in most cancer types, and loss of SIRT4 increases the risk of spontaneous tumors throughout life. SIRT4 participates in regulation of glutamine metabolism by inhibiting GDH expression, and glucose metabolism by inhibiting PDHC expression. SIRT4 is induced by various genetic toxicants, which can regulate cell-cycle progression and genomic fidelity in response to DNA damage. Moreover, oncogenes, tumor-suppressor genes, cancer stem cells, inflammation, and metabolism are the main foci of cancer research. In particular, “metabolic change” has become a new direction for cancer research in recent years. SIRT4 has an important role in suppressing cancer, so SIRT4 could be used as a new biomarker for cancer diagnosis, and to develop targets for new strategies for cancer treatment. Therefore, it is necessary to develop specific pharmacologic activators and inhibitors of SIRT4 that can be used as tools to understand the biology of SIRT4, and which may also be employed as a novel cancer treatment.

## Author Contributions

YB conceived and designed the content of this review. YB wrote the manuscript with the help of JY, YC, FW, CL, and XF. YZ and YY contributed to the final version of the manuscript. All authors contributed to the article and approved the submitted version.

## Funding

This work was supported by the National Natural Science Foundation of China (81672428, 81872427, 81703000), the Applied Technology Research and Development Program of Heilongjiang Province (GA19C002), the Nn10 Excellent Discipline Construction Program (Hepatobiliary and Pancreatic Tumor 2017), and the Fundamental Research Funds for the Provincial Universities of Heilongjiang Province.

## Conflict of Interest

The authors declare that the research was conducted in the absence of any commercial or financial relationships that could be construed as a potential conflict of interest.
